# Bacterial communities in termite fungus combs are comprised of consistent gut deposits and contributions from the environment

**DOI:** 10.1007/s00248-015-0692-6

**Published:** 2015-10-30

**Authors:** Saria Otani, Lars H. Hansen, Søren J. Sørensen, Michael Poulsen

**Affiliations:** Department of Biology, Section for Ecology and Evolution, Centre for Social Evolution, University of Copenhagen, Universitetsparken 15, Building 3, 2100 Copenhagen East, Denmark; Department of Biology, Section for Microbiology, University of Copenhagen, Copenhagen, Denmark; Department of Environmental Science, Aarhus University, Aarhus, Denmark

**Keywords:** Macrotermitinae, Microbiota, *Termitomyces*, 16S rRNA sequencing, Symbiosis

## Abstract

Fungus-growing termites (subfamily Macrotermitinae) mix plant forage with asexual spores of their plant-degrading fungal symbiont *Termitomyces* in their guts and deposit this blend in fungus comb structures, within which the plant matter is degraded. As *Termitomyces* grows, it produces nodules with asexual spores, which the termites feed on. Since all comb material passes through termite guts, it is inevitable that gut bacteria are also deposited in the comb, but it has remained unknown which bacteria are deposited and whether distinct comb bacterial communities are sustained. Using high-throughput sequencing of the 16S rRNA gene, we explored the bacterial community compositions of 33 fungus comb samples from four termite species (three genera) collected at four South African geographic locations in 2011 and 2013. We identified 33 bacterial phyla, with Firmicutes, Bacteroidetes, Proteobacteria, Actinobacteria, and Candidate division TM7 jointly accounting for 92 % of the reads. Analyses of gut microbiotas from 25 of the 33 colonies showed that dominant fungus comb taxa originate from the termite gut. While gut communities were consistent between 2011 and 2013, comb community compositions shifted over time. These shifts did not appear to be due to changes in the taxa present, but rather due to differences in the relative abundances of primarily gut-derived bacteria within fungus combs. This indicates that fungus comb microbiotas are largely termite species-specific due to major contributions from gut deposits and also that environment affects which gut bacteria dominate comb communities at a given point in time.

**Electronic supplementary material:**

The online version of this article (doi:10.1007/s00248-015-0692-6) contains supplementary material, which is available to authorized users.

## Introduction

The Macrotermitinae subfamily of fungus-growing termites lives in an obligate symbiosis with a fungal mutualist in the genus *Termitomyces* [1]. Fungiculture in the Macrotermitinae evolved ca. 30 MYA, and the sub-family members have diversified to nearly 330 described species in 11 genera [38, 44]. The adoption of the fungal ectosymbiont has been central for plant material decomposition [8, 43], allowing this termite lineage to become a major player in plant degradation and nutrient recycling in (sub)tropical Africa and Southeast Asia [2, 3]. For example, up to 90 % of all dry dead wood in Kenya is consumed by species of the Macrotermitinae [4]. This success has been attributed to the complementary mutualistic services of *Termitomyces* [5] and the microbial communities in the termite guts [6–8].

**Fig. 1:**
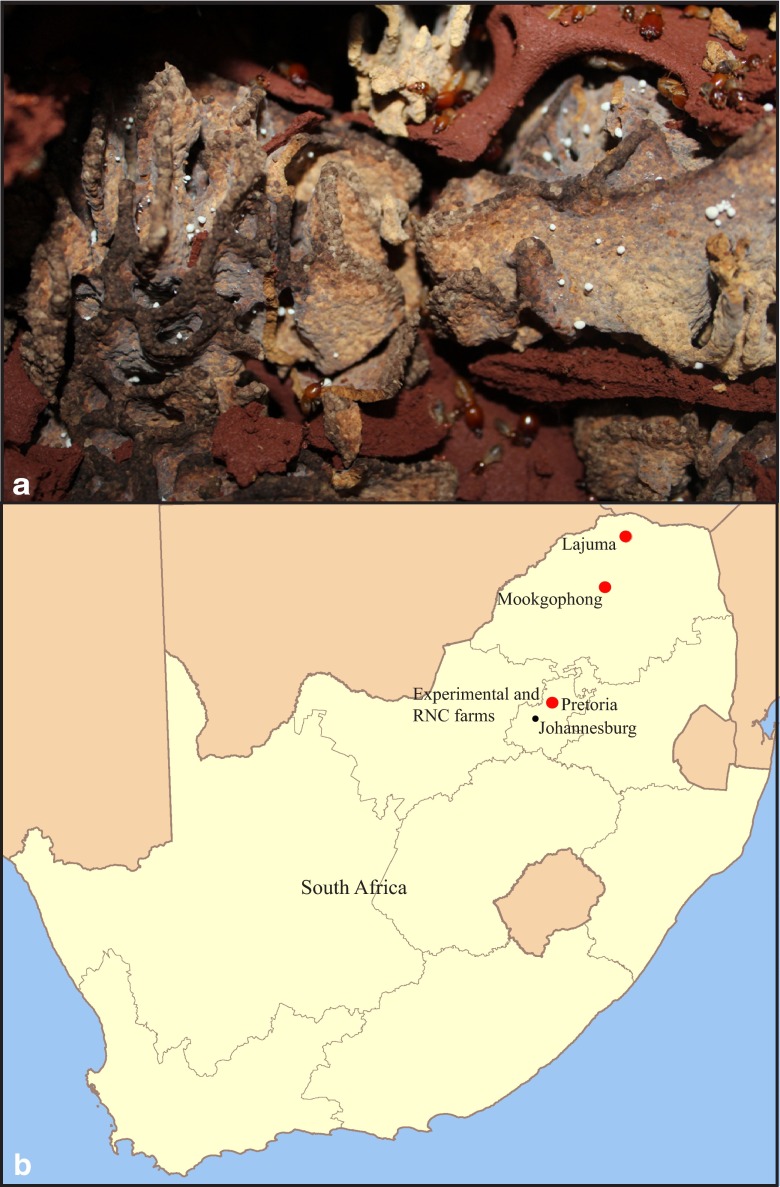
A *Macrotermes natalensis* fungus comb and the four sampling site locations. **a** A fungus comb contains two strata: the darker fresh fungus comb on the top and the lighter older comb below with less plant material and more *Termitomyces* biomass and nodules. **b** A map of South Africa with indications of the four collection sites in *red dots*, two of which were in Pretoria

The most speciose genera of fungus-growing termites are *Macrotermes*, *Odontotermes*, and *Microtermes*, e.g., [5, 23]. Despite their importance, differences in their biology remain poorly understood, particularly in regard to what plant substrate is utilised for fungiculture. It has been proposed that *Macrotermes* species mainly ingest leaf litter [18, 21] and *Odontotermes* primarily wood and leaf litter [19, 20], while species of *Microtermes* have been described to be crop pests and wood feeders [45]. The nests of the three genera are structurally very different: *Macrotermes* mounds are conical and elevated with condensed shelving of fungus chambers on top of each other, with minimal soil separation between chambers (Fig. 1a). *Odontotermes* mounds are flatter and often have ventilation chimneys open to the environment. A main fungus comb is centralised within a deep cavity, and additional satellite fungus chambers surround this main comb [49, 50]. *Microtermes* species build large numbers of sub-spherical chambers that are connected with tunnels and often occur within other termite mounds [49, 50].

Colonies are highly organised with optimised division of labour between colony members to maintain the complex symbiotic system. Generally, a single reproductive pair (a queen and a king) starts the colony. The first workers forage for plant substrate and, in most Macrotermitinae genera, acquire the fungal symbionts from the surrounding habitat [9; exceptions being the genus *Microtermes* and the species *Macrotermes bellicosus*, which have vertical *Termitomyces* transmission]. As the colony matures, different castes are produced; older workers leave the colony to collect plant material, while young workers remain inside the nest [10]. These young workers inoculate the forage material by consuming it along with asexual spores of *Termitomyces* present in nutrient-rich *Termitomyces* nodules [10]. After this first gut passage, the mixture is deposited as a sponge-like structure (the fungus comb) (Fig. 1a) [5, 11]. This creates an upper darker part of the fungus comb that is the termite primary faeces (freshly deposited fungus comb) (Fig. 1a). The lighter (older) fungus comb, where new nodules are produced, is mainly fungal biomass after most plant decomposition has occurred (Fig. 1a) [12, 13]. Older termites consume the oldest parts of the comb during a second gut passage [10, 14], after which only low concentrations of organic material are present in the resulting final faeces [15]. The plant substrate is thus efficiently digested via the combined efforts of fungal and gut bacterial enzymes [8, 16, 17].

Recent molecular approaches has revealed a great diversity of primarily bacteria residing in fungus-growing termite guts [16, 17, 34, 46, 47, 51, 52]. We have recently described what appears to be a core microbiota of the Macrotermitinae: a set of 42 bacterial taxa shared between nine species of fungus-growing termites [17]. This core is distinct from other non-fungus-growing termites [16, 17], but there is variation in the composition of gut microbiotas between fungus-growing termite species, suggesting potential co-adaptation between the termites and gut bacterial communities [17].

Because Macrotermitinae fungus combs are deposited gut contents, bacteria present in the gut could be transferred to the fresh comb. However, gut and fungus comb microbiotas from one macrotermitine species, *Odontotermes formosanus*, were analysed using pyrotag sequencing [24] and showed marked dissimilarities between worker hindgut and comb microbiotas, without detailed comparisons between *O. formosanus* guts and combs and without including comparison to other fungus-growing termite genera. To explore the composition of fungus comb microbiotas in more detail, we sampled 33 combs from three fungus-growing termite genera. Combs were collected from four different sites in South Africa over 2 years, and we used 16S rRNA 454 pyrosequencing to characterise communities. To test the hypothesis that comb communities are shaped by gut deposits, we performed 16S rRNA Illumina MiSeq on worker guts from 25 of the same 33 nests. We determined which gut bacterial taxa are shared with fungus combs and explored whether unique comb taxa or taxa shared with guts have the biggest impact on microbiota fluctuations in comb compositions over time.

## Materials and Methods

### Experimental Material, DNA Extractions, and Termite Species Identification

#### Fungus Combs

Thirty-three fungus combs of three different fungus-growing termite genera were collected from four different sites [Experimental farm (Pretoria), RNC farm (Pretoria), Mookgophong, and Lajuma] in South Africa in February 2011 and February 2013 (Table 1; Fig. 1). In each case, a single apparently healthy comb (fresh and old comb) was collected and stored in RNA*later*^®^ (Ambion, USA). The FastDNA SPIN Kit for Soil (MP Biomedicals, USA) was used for fungus comb DNA extractions following the manufacturer’s instructions, with the addition of two bead beating treatment steps for 25 s each at 6 m/s using a FastPrep^™^ Instrument (MP Biomedicals, USA) after the tissue lysis step, separated by 2 min of incubation on ice. DNA extracts were assessed spectrophotometrically using NanoDrop ND-1000 (Thermo Scientific, Germany).

**Table 1:** Termite colonies, sampling sites, year of collection of the colonies included in this study, the number of high-quality clean 16S rRNA gene sequences, the number of classified genus-level taxa and classified family-level taxa, and Shannon and Simpson diversity indices

Termite species and colony code	Sampling site	Year of collection	Fungus combs (33 colonies)					Termite guts (25 colonies)				
			Total number of sequences	Number of genus-level taxa	Number of family-level taxa	Shannon	Simpson	Total number of sequences	Number of genus-level taxa	Number of family-level taxa	Shannon	Simpson
*Macrotermes natalensis* 115	Experimental farm	2011	12882	138	72	3.46	0.92	13016	200	88	5.32	0.99
*Macrotermes natalensis* 116	Experimental farm	2011	8411	131	81	3.39	0.92	12682	174	78	5.34	0.99
*Macrotermes natalensis* 118	Mookgophong	2011	4949	58	34	2.93	0.91	12035	155	79	5.33	0.99
*Macrotermes natalensis* 132	Mookgophong	2013	8518	190	110	3.56	0.94	14906	195	90	5.52	0.99
*Macrotermes natalensis* 133	RNC farm	2013	4239	109	79	3.25	0.87	12416	195	82	5.55	0.99
*Macrotermes natalensis* 134	RNC farm	2013	7062	104	74	3.22	0.88	12639	170	65	5.05	0.99
*Macrotermes natalensis* 135	RNC farm	2013	6700	174	107	3.67	0.93	13035	173	79	5.34	0.99
*Macrotermes natalensis* 136	Lajuma	2013	6519	91	58	2.61	0.78	15084	185	78	5.33	0.99
*Macrotermes natalensis* 138	Lajuma	2013	5551	110	76	3.14	0.87	12140	178	69	5.41	0.99
*Macrotermes natalensis* 141	Lajuma	2013	6978	105	65	2.56	0.81	15847	207	88	5.52	0.99
*Microtermes* sp. 115-1	Experimental farm	2011	7018	45	37	2.26	0.82	NA	NA	NA	NA	NA
*Microtermes* sp. 115-2	Experimental farm	2011	4615	71	59	2.46	0.83	NA	NA	NA	NA	NA
*Microtermes* sp. 115-3	Experimental farm	2011	8924	66	51	2.27	0.79	NA	NA	NA	NA	NA
*Microtermes* sp. 127	Experimental farm	2013	20100	316	162	4.20	0.96	15665	128	38	4.80	0.98
*Microtermes* sp. 130	Mookgophong	2013	7536	248	151	4.49	0.97	10847	123	48	4.76	0.98
*Microtermes* sp. 132	Mookgophong	2013	10705	357	174	4.79	0.98	NA	NA	NA	NA	NA
*Microtermes* sp. 135	RNC farm	2013	9582	256	154	4.51	0.98	11338	131	38	5.01	0.99
*Microtermes* sp. 140	Lajuma	2013	10186	292	141	4.74	0.98	15985	140	44	5.06	0.99
*Microtermes* sp. 143	RNC farm	2013	13155	350	185	4.86	0.98	16265	118	42	4.17	0.96
*Odontotermes* cf. *badius* 111	Experimental farm	2011	22751	281	143	3.86	0.95	11129	146	57	5.09	0.99
*Odontotermes* cf. *badius* 112	Experimental farm	2011	26716	280	134	3.99	0.96	13515	138	43	5.11	0.99
*Odontotermes* cf. *badius* 114	Experimental farm	2011	5447	136	53	3.77	0.94	14611	136	47	4.74	0.98
*Odontotermes* cf. *badius* 122	RNC farm	2011	6487	109	68	3.40	0.91	14596	138	43	5.06	0.99
*Odontotermes* sp. 120	RNC farm	2011	12869	231	116	3.62	0.93	NA	NA	NA	NA	NA
*Odontotermes* sp. 121	RNC farm	2011	15339	249	108	4.10	0.96	NA	NA	NA	NA	NA
*Odontotermes* sp. 124	Experimental farm	2011	20322	262	108	3.63	0.95	NA	NA	NA	NA	NA
*Odontotermes* sp. 125	Experimental farm	2011	7955	59	30	2.19	0.81	NA	NA	NA	NA	NA
*Odontotermes* cf. *badius* 126	Experimental farm	2013	15298	354	168	4.54	0.98	13435	153	53	5.13	0.99
*Odontotermes* sp. 127	Experimental farm	2013	4460	189	125	3.96	0.94	15414	139	48	5.12	0.99
*Odontotermes* sp. 128	Experimental farm	2013	13913	295	140	4.06	0.95	20926	154	60	5.11	0.99
*Odontotermes* sp. 129	Mookgophong	2013	9199	236	131	3.71	0.94	18051	166	65	5.28	0.99
*Odontotermes* sp. 130	Mookgophong	2013	12334	332	150	4.13	0.95	34702	158	62	4.87	0.98
*Odontotermes* sp. 143	RNC farm	2013	15496	372	165	3.99	0.94	47763	157	43	5.20	0.99

#### Termite Guts

Twenty-five colonies from the 33 that were collected for the fungus combs were also used for termite sampling (Table 1, Fig. 1). Ten whole guts from major workers were dissected and pooled from each colony. The DNeasy Blood and Tissue Kit (Qiagen, Germany) was used for DNA extractions following the manufacturer’s instructions. DNA yields were assessed spectrophotometrically using NanoDrop ND-1000 (Thermo Scientific, Germany).

### *Odontotermes* Identification and Phylogenetic Analysis

For *Odontotermes* colonies, in which molecular species determination was necessary due to uncertainty in morphological identification [26], workers and soldiers were collected and stored in 96 % ethanol. This was not necessary for *Macrotermes natalensis*, because only a single *Macrotermes* species exists in this area [25], and was not done for *Microtermes*, because termites could not be obtained for *Microtermes* samples from 2011. The DNeasy Blood and Tissue Kit (Qiagen, Germany) was used after a homogenisation step to extract DNA from *Odontotermes* termite heads. PCR was performed on 14 *Odontotermes* DNA extracts to amplify the mitochondrial cytochrome oxidase II gene (COII) to obtain a barcode that could be compared to available sequences in GenBank. Reactions were prepared in 25 μl final volume using A-tLeu forward primer (5′-ATG GCA GAT TAG TGC AAT GG-3′) and B-tLys reverse (5′-GTT TAA GAG ACC AGT ACT TG-3′) [27, 28]. The PCR mixture contained 8.5 μl sterile distilled water, 1 μl of each primer, 2 μl template, and 12.5 μl REDTaq ReadyMix (Sigma-Aldrich, USA). The conditions for PCR were 98 °C for 30 s followed by 35 cycles of 94 °C for 30 s, 50 °C for 30 s, and 72 °C for 30 s with a final extension step at 72 °C for 4 min. Target PCR products were visualised by agarose gel electrophoresis and purified using MSB Spin PCRapace (STRATEC Molecular, Germany). The samples were subjected to sequencing at Eurofins MWG Operon (Ebersberg, Germany). Resulting sequences were aligned in Geneious 6.1.6 using the MUSCLE algorithm [29], and a Neighbour Joining tree, including the 14 *Odontotermes* colonies involved in the present study, two additional colonies, and *Odontotermes* COII sequences available from GenBank, was generated in TreeView [30] with Kimura two-parameter estimates. Sequences generated as part of this study have been deposited in GenBank (accession numbers KJ4590682–KJ4590697).

### Bacterial Community Characterisations in Fungus Combs

#### Fungus Comb PCR Amplification and 454 Pyrotag Sequencing

The 16S rRNA gene was amplified using the primers 341F (5′-CCTACGGGRBGCASCAG-3′) and 806R (5′-GGACTACNNGGGTATCTAAT-3′) flanking the hypervariable V3–V4 regions [31]. The primers were modified by adding sample-specific multiplex identifier barcodes (MID) (5′-Adaptor A) to the forward primer and a universal sequence (5′-Adaptor B) to the reverse primer. The amplification reaction was prepared in 20 μl final volume containing the following: 12.4 μl sterile distilled water, 0.4 μl dNTPs (10 μM), 4 μl 5× HF buffer, 1 μl of each primer, 1 μl template, and 0.2 μl Phusion High-Fidelity DNA Polymerase (Thermo Scientific, Germany). The conditions for PCR were 98 °C for 30 s followed by 15 cycles of 98 °C for 5 s, 56 °C for 20 s, and 72 °C for 20 s with a final extension step at 72 °C for 5 min. This reaction was done after a previous amplification using the same primers and conditions but with 30 PCR cycles. The final target PCR products were visualised by agarose gel electrophoresis, then extracted and purified from the gel using Montage DNA Gel Extraction Kit (Millipore Corporation, USA). DNA concentrations were quantified using Quant-iT dsDNA High-Sensitivity Assay Kit and Qubit fluorometer (Invitrogen). The samples were subjected to sequencing on GS FLX Titanium PicoTiterPlate using a GS FLX Titanium Sequencing Kit according to the manufacturer’s instructions (Roche).

#### Sequence Filtering and Taxa Classification

The raw flowgrams were fed into MOTHUR (version 1.31.2, [32]), where multiplexed reads were assigned to samples based on unique barcodes and erroneous reads were removed by denoising. Sequences containing ambiguous bases (N), with mismatches to the 16S rRNA primers, homopolymer stretches longer than 10 bases, or sequences shorter than 200 bp, were excluded from subsequent analyses during several filtering steps in MOTHUR. Clean sequences were submitted to the Sequence Read Archive (SRA) in GenBank (accession numbers SRR1293514–SRR1293516, SRR1293655, SRR1293679, SRR1293686, SRR1293696–SRR1293703, SRR1293771, SRR1293794, SRR1293811–SRR1293813, SRR1293816–SRR1293818, SRR1293820–SRR1293825, SRR1293827, SRR1293828, SRR1293831, SRR1293837, SRR1293845). High-quality sequences were aligned against the SILVA 102 non-redundant database using MOTHUR. Alignments were subsequently assigned to taxa using the naïve Bayesian classifier with a confidence threshold of 60 % and a manually curated reference database DictDb v. 2.3 [33]. This database was generated from the SILVA database with additional termite and cockroach gut 16S rRNA gene sequences added to improve the classification resolution; it is available upon request. Rarefaction curves based on 97 % sequence similarity cutoff were generated using R [40].

#### Comparative Analyses of Community Diversity and Similarity

The representative clusters were sorted according to the genus-level classifications, and taxa abundances were calculated as the number of reads per taxon. R [40] was used to calculate community similarities; principal coordinates analysis (PCoA) was performed based on the Bray-Curtis index to determine community similarity between all 33 samples. Three additional PCoA analyses were performed on samples from within each of the three termite genera.

### Bacterial Community Characterisations in Termite Guts

#### Termite Worker Gut PCR Amplification and MiSeq Sequencing

The V4 region of the 16S rRNA gene was amplified using the primers v4.SA504 (5′-AATGATACGGCGACCACCGAGATCTACACCTGCGTGTTATGGTAATTGTGTGCCAGCMGCCGCGGTAA-3′) and v4.SB711 (5′-CAAGCAGAAGACGGCATACGAGATTCAGCGTTAGTCAGTCAGCCGGACTACHVGGGTWTCTAAT-3′). The V4 region amplification was done using a dual-indexing sequencing strategy (41), and the PCR mixture was prepared in 20 μl volume containing 11.85 μl sterile distilled water, 2 μl of each primer (4.0 μM), 2 μl of 10× AccuPrime PCR buffer II (Life Technologies, USA), 1 μl DNA template, and 0.15 μl AccuPrime High Fidelity Taq DNA polymerase (Life Technologies, USA). PCR conditions were 95 °C for 2 min followed by 30 cycles of 95 °C for 20 s, 55 °C for 15 s, and 72 °C for 5 min followed by 72 °C for 10 min (42). Library normalisation was done using Life Technologies SequalPrep Normalization Plate Kit (Life Technologies, USA) following the manufacturer’s instructions. Sample concentration was measured using Kapa Biosystems Library Quantification kit for Illumina platforms (Kapa Biosystems, USA). The size of the library amplicons was determined using the Agilent Bioanalyser High Sensitivity DNA analysis kit (Invitrogen). The samples were subjected to sequencing on the Illumina MiSeq platform using MiSeq Reagent Kit V2 500 cycles (Illumina) [41, 42].

#### Sequence Filtering and Taxa Classification

Sequence analysis was performed using MOTHUR (version 1.34.3, [32]), and the standard operating procedure (SOP) was followed as described at http://www.mothur.org/wiki/MiSeq_SOP [41]. Briefly, the paired end reads were assembled into contigs and then sequences were subjected to several filtering steps. Clean reads were submitted to SRA, GenBank (accession numbers SRR2085096–SRR2085120). High quality sequences were aligned against the SILVA 102 database. Alignments were afterwards assigned to taxa with a confidence threshold of 80 %, and operational taxonomic units (OTUs) were calculated at 3 % species level classification. Finally, rarefaction curves based on 97 % sequence similarity cutoff were generated using R [40].

#### Comparative Analyses of Gut Community Diversity and Similarity

Relative abundances of identified taxa were calculated as the number of reads per taxon for the 25 gut communities. PCoA was performed in R [40] based on the Bray-Curtis index to determine community similarity between the 25 samples from the four termite species from the three termite genera (*M. natalensis*, *Microtermes* sp., and *Odontotermes* cf. *badius* and *Odontotermes* sp.).

#### Comparison of Comb and Gut Community Compositions

To investigate bacterial community similarities between the guts and fungus combs, we identified overlapping and unique taxa in each of the 25 colonies with sequencing data from both worker guts and fungus combs. Quantitative contributions of the overlapping taxa were calculated as the proportion of reads that were assigned to overlapping versus unique taxa in guts and combs. The use of different sequencing technologies and primer sets to amplify the V4 region of the 16S rRNA gene in fungus combs (454 titanium sequencing) and guts (Illumina MiSeq) precluded comparisons to the genus level, so all comparisons were performed at the family level. Gut and comb family bacterial taxa were compared manually and separately for each of the 25 tested colonies, and identical taxonomical names were considered shared taxa. Sequence comparisons were not possible due to length differences between 454 and MiSeq sequences (http://www.mothur.org/).

To evaluate whether shared or unique bacterial taxa contributed the most to similarities between communities within and between termite species across years, we performed four additional PCoAs. Two of these PCoAs compared fungus comb communities: one included only taxa that were also present in guts and the other included only taxa that were unique to combs. The remaining two PCoAs compared gut communities: one included taxa that were also present in combs and the other included only taxa that were unique to guts.

## Results

### *Odontotermes* Phylogenetic Analysis

COII genes were successfully amplified from the 14 *Odontotermes* samples, in addition to two samples not included in this study. BLAST (Table S1) and phylogenetic analysis (Fig. S1) of the 390-bp fragment suggested that we had collected five *Odontotermes* cf. *badius* and nine *Odontotermes* sp. colonies from a well-supported phylogenetic clade most closely related to *Odontotermes* sp. and *Odontotermes latericeus* (Fig. S1). Therefore, combs from this species are hereafter labelled *Odontotermes* sp.

### Pyrosequencing Data from Fungus Combs

We obtained between 4239 and 26,716 high-quality reads (average ± SE 10,673 ± 977) per comb sample (Table 1). The resulting rarefaction curves were approaching saturation for most samples, indicating that coverage in general was sufficient for community structure analyses. Even though the rarefaction curves for the samples *Odontotermes* sp. 127, *Odontotermes* sp. 129, and *Microtermes* spp. 135 indicated that additional sequencing would be required to cover the expected bacterial diversity (Fig. S2), they were included in the analyses. The number of bacterial genera ranged between 58 and 372 (average 196) (Table 1), with *M. natalensis* combs harbouring the least (*M. natalensis* 121, *Microtermes* sp. 222, *Odontotermes* sp. 229, and *O.* cf. *badius* 232). Samples collected in 2011 had fewer bacterial taxa, particularly for *Microtermes* sp., while sampling site did not appear to influence the observed number of taxa in combs (Table 1). Shannon and Simpson diversity indices were similar across the comb samples with slightly higher values in *Microtermes* comb communities from 2013 (Table 1).

### Taxonomic Composition of Comb Bacterial Communities

Using the termite-specific improved SILVA database (DictDb) allowed relatively high classification success in all samples (54.5–76.8 % at the genus level; Table 1). Thirty-three bacterial phyla were identified in the 33 combs, and the five most abundant phyla accounted for 92.4 % of the total number of reads (Firmicutes, Bacteroidetes, Proteobacteria, Actinobacteria, and Candidate division TM7) (Fig. S3, Table S2). Among the most abundant phyla, Actinobacteria were more abundant in *Microtermes* sp. compared to *M. natalensis* and *Odontotermes*, whereas Firmicutes and Bacteroidetes were less abundant in *Microtermes* sp. (Fig. S3). Among the less abundant phyla, Acidobacteria and Chloroflexi were more abundant in *Microtermes* sp. from 2013 compared 2011 and to the other termite genera. Spirochaetes were not abundant in combs, representing on average only 1.2 % of the total classified reads (Fig. S3, Table S2).

Most bacterial genera were unevenly distributed across combs. An overview of all 1795 genus-level taxa obtained in the analysis can be found in the interactive Table S2, which provides read percentages for each bacterial taxon from phylum to genus level for each comb sample. Of these genus-level taxa, 1387 were bacteria, and the 100 most abundant bacteria across all 33 combs are presented in Fig. S5 as a heatmap of relative abundances. Among these, only a few taxa were present in relatively high abundance across all comb samples; for example, *Alistipes* (13.3 % average abundance across all combs), BCf917 termite group in the family Rikenellaceae (3.6 %) and *Herbaspirillum* 1 in the Oxalobacteraceae (2.2 %). A number of other genera were abundant on average, but absent or in low abundance in some combs; for example, uncultured 1 in the family Corynebacteriaceae (6.1 %), uncultured 3 in the family Peptostreptococcaceae (3.9 %), Gut Cluster 1 in the family Ruminococcaceae (2.3 %), Incertae Sedis 2 in the Planococcaceae (2 %), Gut Cluster 2 in the family Lachnospiraceae (1.9 %), “*Candidatus Arthromitus*” (1.7 %) and Incertae Sedis 6 (Planococcaceae 2) (1.6 %) (Table S2). Despite the presence of several abundant taxa in the communities, the appreciable difference between the 2 years of collection led to a large variation in relative abundances between fungus combs within and between genera (Table S2, Fig. S5). For example, the dominant taxon *Alistipes* was less abundant in *Microtermes* combs in 2011 compared to 2013, uncultured 1 (Corynebacteriaceae) was present only in *M. natalensis* and *Microtermes* sp. samples from 2011 (particularly *M. natalensis* 118), and uncultured 3 (Peptostreptococcaceae) was only detected in three samples (*M. natalensis* 118, *Odontotermes* sp. 124, and *Odontotermes* sp. 125) from 2011 (Fig. S5, Table S2). Similarly, Gut Cluster 2 and “*Candidatus Arthromitus*” were detected in high abundance only in 2013, while *Herbaspirillum* 1 was only abundant in *M. natalensis* and *Microtermes* sp. from 2011. The BCf917 termite group was relatively abundant in *M. natalensis* samples in 2013, less in *Odontotermes* in 2011, and in very low relative abundance in the remaining samples (Fig. S5, Table S2). Incertae Sedis 2 and Insertae Sedis 6 showed high relative abundances only in *M. natalensis* 118, *Odontotermes* sp. 124, and *Odontotermes* sp. 125 from 2011 (Fig. S5, Table S2).

### Comb Ordination Analyses

**Fig. 2:**
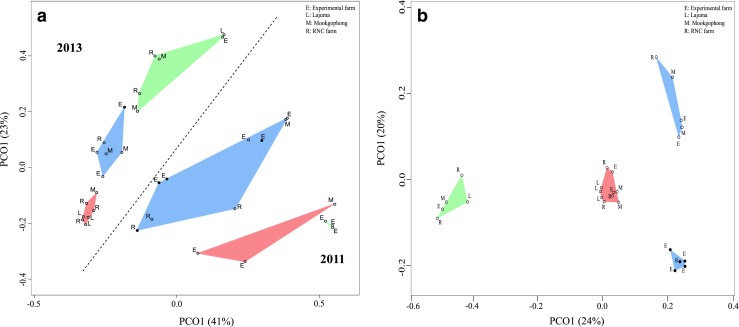
**a** PCoA similarity analysis of the 33 fungus comb samples visualised via Bray-Curtis distances across samples. Fungus comb samples from the same host taxa are connected and have the same colour (*red*: *Macrotermes natalensis*, *blue*: *Odontotermes*, *solid circles* are *O*. cf. *badius* and *open circles* are *Odontotermes* sp., and *green*: *Microtermes* sp.). Sampling locations are indicated, and the dashed line separates the sampling years 2011 and 2013. **b** PCoA similarity analysis of the 25 gut samples visualised via Bray-Curtis distances across samples. Gut samples from the same host taxa are connected and *coloured* as in **a**

We visualised Bray-Curtis distances between communities in four different PCoAs: the entire data set (33 samples, Fig. 2a), *M. natalensis* (10 fungus combs, Fig. S4B), *Microtermes* sp. (9 fungus combs, Fig. S4C), and *O.* cf. *badius* and *Odontotermes* sp. (14 fungus combs, Fig. S4A). There was a distinct separation in all PCoAs between years (Figs. 2a and S4), which was also evident for individual termite genera (Fig. S4A–C). Community compositions were secondarily influenced by termite species origin, with bacterial communities of combs collected from a termite species being more similar to each other than to samples collected from other termite species in the same year (Fig. 2a). Within termite species, there was also an apparent effect of sampling site; for example, the two *Odontotermes* sp. colonies collected at the RNC farm in 2011 were distant from the remaining colonies of *Odontotermes* sp. collected at other locations (Fig. S4A).

The *O.* cf. *badius* 122 comb community was more similar to samples from the other *Odontotermes* species from the same sampling site (RNC farm) than to samples from other sites (Fig. S4A). *M. natalensis* showed the same trend, with *M. natalensis* 115 and 116 fungus combs collected in 2011 at the Experimental farm being more similar to each other than to *M. natalensis* 118 collected the same year in Mookgophong (Figs. 2a and S4B), and Lajuma-collected *M. natalensis* samples from 2013 clustering closer to each other than to communities from other sites (Figs. 2a and S4B). However, some combs from the same termite species (e.g. Mn138 and Mn134, Od128, and Od130) were similar in composition despite being from geographically distinct sites up to 450 km apart. A spatial effect of sampling site did not appear for *Microtermes* fungus comb communities, probably due to the limited number of samples from 2011 (Table 1, Fig. S4C).

### Illumina MiSeq Data from Worker Guts

16S rRNA gene sequencing of the guts generated between 10,847 and 47,763 high-quality reads (average ± SE 16,321 ± 1612) per gut sample (Table 1), and rarefaction analysis showed sufficient coverage of bacterial communities (Fig. S6). A total of 5178 unique OTUs at the 3 % cutoff level were identified after filtering and sequence analysis of gut communities, with 425 to 860 OTUs per gut sample (Table 1).

### Taxonomic Composition of Gut Bacterial Communities

Gut community reads were classified using the publicly available SILVA database, and 27 bacterial phyla were identified in the 25 termite guts (Fig. S7). We were not able to classify the gut sequences using the termite specific DictDb v. 2.3 database, which was used for the comb reads, as it was developed for 454 pyrosequencing analyses. The most abundant phyla were Bacteroidetes, Firmicutes, Spirochaetes, Proteobacteria, and Planctomycetes, which accounted for 93.5 % of all sequence reads (Fig. S7, Table S3). No differences in phylum relative abundances were noticed between the samples from different years (2011 and 2013), whereas differences were apparent between guts from different termite genera (Fig. S7). For example, Spirochaetes was more abundant in *Microtermes* and *Odontotermes* compared to *Macrotermes* (Table S3) and Planctomycetes was less abundant in *Microtermes* guts compared to the other two genera (Table S3). Members of Actinobacteria were generally low in abundance and accounted for only 2.1 % of the total number of reads.

Genus-level OTU classification identified 495 bacterial genera from the 25 gut communities. An interactive table of all generated OTUs and their assigned taxa is presented in Table S3 with the corresponding relative abundances. A heatmap of the 100 most abundant gut bacteria is presented in Fig. S8. *Alistipes* was the most abundant (on average 13.2 % across all guts), followed by *Treponema* (9.1 %), and *Dysgonomonas* (7.1 %). Unlike comb communities, relative abundances of bacterial taxa were not different between 2011 and 2013 gut samples (Fig. S8, Table S3), whereas differences were apparent between termite host genera and species. For example, *Treponema* was more abundant in *Odontotermes* and *Microtermes* guts (on average 6.3 and 3.6 %, respectively) compared to *Macrotermes* guts (1.2 %) (Fig. S8, Table S3), and the genus *Parabacteroides* was high in abundance in *Odontotermes* guts (on average 3.4 %), particularly in *O*. cf. *badius*, compared to *Macrotermes* (0.2 %) and *Microtermes* (0.07 %) (Fig. S8, Table S3). *Tannerella* was abundant in *O.* cf. *badius* guts (2.9 %), but almost absent in *Odontotermes* sp. guts (0.002 %) (Fig. S8, Table S3).

### Gut Ordination Analyses

Gut microbiota similarities were assessed by Bray-Curtis distances and in PCoA plots (Fig. b). A clear signal of termite species was present in gut communities, where microbiotas within a termite species were more similar to each other than to other termite species (Fig. 2b). In contrast to comb communities, there was no effect of year on gut bacterial composition (Fig. 2b).

### Comparison of Comb and Gut Community Compositions

**Fig. 3:**
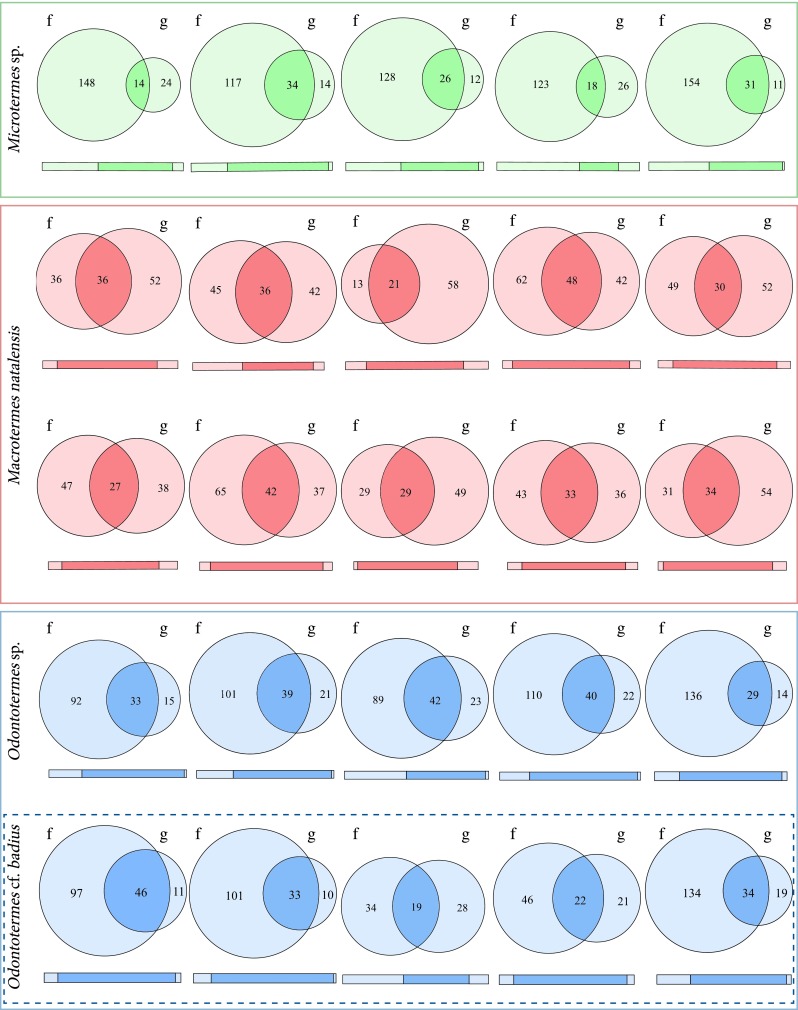
Venn diagrams of shared fungus comb and gut microbiota families in the 25 colonies where samples for both could be sequenced. *f* are the fungus comb communities and *g* are the gut communities. Colonies from the same host taxa have the same *colour* (*red*: *Macrotermes natalensis*, *green*: *Microtermes* sp., and *blue*: *Odontotermes* spp. (*O*. cf. *badius* within *dashed box*)). The *numbers* represent the number of family-level bacterial taxa identified in the fungus combs only, in the guts only, or shared between the two communities. The *bars* represent the abundances of the high-quality reads that were assigned to bacterial families presented in the Venn diagrams in each colony (*light colour*: proportion of reads that are unique to guts or fungus combs, *dark colour*: proportion of reads shared by the two communities). Detailed numbers are presented in Table S4

The comparison between gut-associated and comb-associated microbiotas was done separately for each of the 25 colonies (Fig. 3). There were between 14–48 bacterial families overlapping between the guts and fungus combs in the 25 colonies (Fig. 3). In *Microtermes*, 36.8–73.8 % of families identified in guts were present in combs, 26.6–53.3 % of gut families were present in *Macrotermes* combs and 40.4–80.7 % of *Odontotermes* gut families were present in combs. Smaller proportions of families identified in the combs were present in guts as follows: 8.6–22.5 % in *Microtermes*, 36.5–61.8 % in *Macrotermes*, and 17.6–35.9 % in *Odontotermes* (Venn diagrams in Fig. 3; Table S4). Thus, *Microtermes* and *Odontotermes* colonies had more overlapping families between guts and fungus combs and contained relatively few unique gut families compared to *M. natalensis* (Fig. 3). The proportion of reads belonging to overlapping families ranged from 64.6 to 98 % out of the total number of gut reads across colonies (64.6–97.7 % in *Microtermes*, 79.7–92.5 % in *Macrotermes*, and 77–98 % in *Odontotermes*), and 31.9–96.3 % of the total number of fungus comb reads across colonies were shared with guts (31.9–73.7 % in *Microtermes*, 58.5–96.3 % in *Macrotermes*, and 51.9–89.5 % in *Odontotermes*) (*bars* in Fig. 3, Table S4). Thus, while the reads of shared families dominated gut communities in all termite genera, the proportion of shared comb reads varied by termite genus. In *M. natalensis*, shared reads dominated both gut and comb communities, while larger portions of *Microtermes* and *Odontotermes* comb communities contained reads from families not present in gut communities (*bars* in Fig. 3, Table S4).

**Fig. 4:**
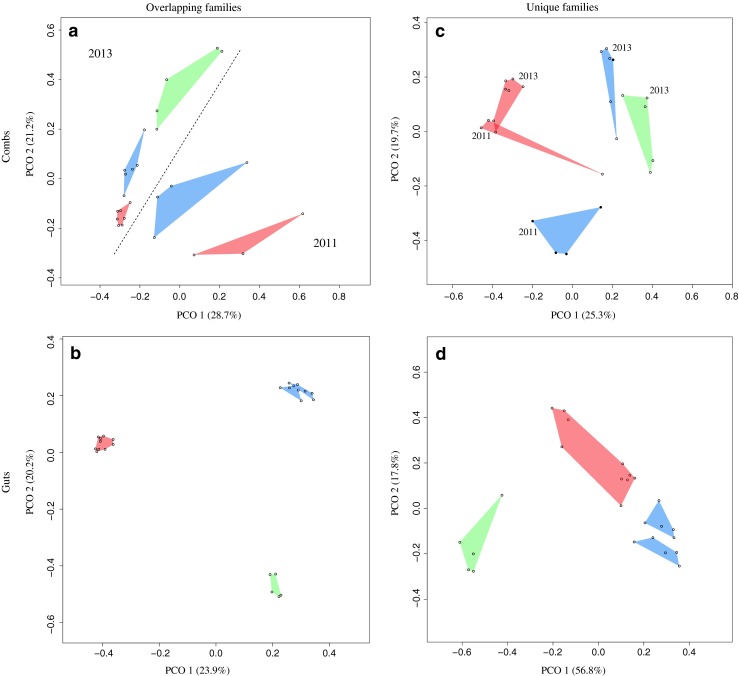
PCoA similarity analyses visualising Bray-Curtis distances between fungus comb microbiotas (**a, c**) and gut microbiotas (**b**, **d**) when measuring distances between fungus comb bacterial communities that overlap with the gut bacteria (**a**), distances between fungus comb bacterial communities that are unique to combs (**c**), distances between gut bacterial communities that overlap with fungus comb bacteria (**b**), and distances between gut bacterial communities that are unique to the guts. Samples from the same host taxa are connected and have the same *colour* (*red*: *Macrotermes natalensis*, *green*: *Microtermes* sp., *blue*: *Odontotermes* spp., *solid circles* are *O*. cf. *badius* and *open circles* are *Odontotermes* sp.)

To investigate whether unique or overlapping bacteria contributed the most to the differences between fungus combs across years, Bray-Curtis distances (including bacterial relative abundance) were calculated and visualised in four PCoAs that included taxa in (1) comb communities that overlapped with guts (Fig. 4a), (2) gut communities that overlapped with combs (Fig. 4b), (3) comb communities that did not overlap with guts (Fig. 4c), and (4) gut communities that did not overlap with combs (Fig. 4d). The first of these (Fig. 4a) showed the same temporal separation between years as the complete fungus comb microbiotas (Figs. 2a and 4a). Similarly, analysis of the gut bacteria overlapping with fungus combs separated in PCoA space (Fig. 4b) in a similar pattern as when analysing the entire gut microbiotas in Fig. 2b, except that the two *Odontotermes* spp. were close in PCoA space. These patterns of community similarities were diluted when only unique gut or comb bacteria were included in the analyses (Fig. 4c, d). Nevertheless, comb communities remained largely separated by year, except in *Macrotermes* (Fig. 4c), while gut communities remained separated by termite species, but with substantially increased variation within species (Fig. 4d).

## Discussion

We present the first detailed comparison of bacterial communities in guts and fungus combs of fungus-growing termites. By comparing 33 different comb microbiotas from within and between species from three termite genera over 2 years, we found that communities were often very similar within termite species when obtained from the same year, but that there were marked shifts in comb community compositions across years of sampling. In contrast, analyses of guts from 25 of the 33 colonies showed that gut communities were consistent in composition within termite host species over time. Comparisons of comb and gut communities showed that (i) a proportion of bacterial families are shared between guts and fungus combs, (ii) in most colonies, the majority of reads in comb communities belong to families shared with gut microbiotas, (iii) unique families are present in both guts and combs, but these represent a minority of sequence reads, and (iv) although the above patterns were true across the three termite genera (four species) tested, there were consistent differences between termite species. Below, we discuss the implications of these findings, focusing on how bacterial communities of fungus combs are assembled.

### Shared Bacteria Across Combs and Guts

Comb communities were dominated by bacteria that were also present in termite guts (Fig. 3). Because gut and fungus comb communities were sequenced using two different sequencing platforms, different primer sets, bioinformatics analyses [41] and reference databases, detailed genus-level community analyses were not possible and comparisons were restricted to higher phylogenetic levels. However, checking the two sets of primers using the SILVA TestPrime online tool (http://www.arb-silva.de/search/testprime/) indicated that they should not be biased towards or against the major phyla identified in either of the two analyses.

Firmicutes and Bacteroidetes dominated both guts and fungus combs and accounted for 60 % of all reads, which is comparable to previous reports on fungus-growing termite guts [16, 17]. These phyla include members that are important for carbohydrate metabolism, reductive acetogenesis, and fungal cell wall degradation [43, 48]. Actinobacteria were more abundant in combs compared to guts (Figs. S3 and S7), suggesting that growth conditions for Actinobacteria are better in combs than in guts. It could also indicate that they are introduced to the comb from the surrounding soil, where Actinobacteria are abundant [52]. It has been suggested that Actinobacteria may play defensive roles in the symbiosis by suppressing unwanted microorganisms [39]. If this is so, our findings suggest that this function may be more important in fungus combs than in termite guts. The remaining phyla were similar in relative abundances between the guts and combs (Figs. S3 and S7).

A comparison of guts and combs at the level of bacterial families (Fig. 3) showed similar overall patterns of shared communities across the four species (three genera) of termites. Large fractions of sequence reads were consistently shared between combs and guts within colonies (*bars* in Fig. 3), implying that when fresh comb is built through gut deposits of fragmented substrate inoculated with *Termitomyces*, this is accompanied by the deposition of a substantial diversity of gut bacteria. These deposits are likely to contain a large proportion of termite gut symbionts, but it is conceivable that they may also include substrate microorganisms that pass through the termite gut. Although we cannot distinguish between these two sources, the former is likely to be the most important, as fungus combs contain the dominant gut symbionts of fungus-growing termites.

Previous studies have suggested that diet may influence the composition of termite gut microbiota [35, 36], and different fungus-growing termite genera tend to use different substrates for fungiculture [18–22]. Our finding of consistent gut microbiota compositions over time and geographical distances suggests that gut communities are resilient to changes in substrate microbial content; however, different substrates may transport different bacteria to the combs if these bacteria remain low in abundance within guts, but proliferate after deposition in combs. At present, substrate use and the microbial content of substrates remain poorly characterised, and further work will be needed to establish which bacteria are potentially delivered to combs through the substrate.

Although most sequence reads belonged to shared bacterial families, several taxa were unique in guts or fungus combs. Unique gut taxa are likely to be symbionts that are not deposited with the fresh comb, e.g. symbionts in low abundance within guts or symbionts that are tightly associated with the termite gut wall and absent in the lumen. Unique comb taxa are likely to be from the surrounding soil or substrate-dwelling bacteria that pass the gut and only proliferate within combs. In a recent study, Makonde et al. [52] detected increased relative abundances of Actinobacteria in the mounds and savannah soils compared to termite guts in two fungus-growing termite genera. However, since soil or mound samples were not available for comparison in this study, we could not test their contributions to the identified unique bacterial taxa in combs.

### Factors Contributing to Differences in Comb Communities Between Termite Genera

Both gut and comb communities consistently cluster according to termite host species (Fig. 2a, b), making termite taxon the main predictor of comb community structure. The differences in gut microbiotas between termite species, and particularly termite genera [16, 17, this study], and the presence of many gut taxa in fungus combs imply that differences in comb microbiotas between termite genera are shaped by differences in gut deposits between termite genera (Fig. 2).

The variation in the shared bacterial communities between guts and fungus combs appears to be related to variation in the total number of bacterial families in the guts and fungus combs between the termite genera. For example, *Macrotermes* colonies harboured the highest number of gut families (on average 80), but the lowest number of comb bacterial families (on average 76) (Table 1). It is likely due to this variation between *Macrotermes* gut and comb bacterial counts that fewer gut families are shared with fungus combs, while more comb families are present in guts compared to the other two termite genera (Table 1, Fig. 3). In contrast, most gut families in *Microtermes* and *Odontotermes* were also present within combs, but fewer fungus comb families were present in guts (Fig. 3, Table S4). However, when adding the number of sequence reads assigned to the families shared between guts and fungus combs, most reads from *Macrotermes* guts and combs are from shared bacterial families (*bars* in Fig. 3, Table S4, see “Results” section), while *Microtermes* and *Odontotermes* share most gut sequence reads with fungus combs, and relatively smaller proportions of their fungus comb reads overlap with guts (particularly in *Microtermes*) (*bars* in Fig. 3, Table S4).

### Factors Contributing to Differences Over Time Within Genera

The ordination analysis of the comb microbiota results showed that communities were particularly different across years, which was consistent across the three termite genera (Figs. 2a and S4). However, several comb communities from geographically distant sites (>450 km apart) were remarkably similar within a year (e.g. *M. natalensis* 138 and *M. natalensis* 134 and *Odontotermes* sp. 128 and *Odontotermes* sp. 130; Figs. 2a and S4). Given that the majority of bacterial sequences identified within combs were shared with gut communities, we expected that comb communities would strongly mirror gut communities. The latter, however, were consistent in composition over time.

We were not able to quantitatively explore community similarities between combs and guts in PCoA space due to the use of different sequencing techniques (Fig. 2), but additional PCoA analyses suggested that both changes to the relative abundances of bacterial families shared with gut communities (Fig. 4a) and the sets of bacteria unique to combs (Fig. 4c) contribute to the separation of fungus comb microbiotas from the same termite species across the 2 years of sampling. The analyses did, however, suggest that the set of shared bacteria might have a larger impact on the temporal variation (Fig. 4a).

The factors contributing to community changes over time in a manner that leads to consistent communities within termite species within years are yet to be fully addressed. Nevertheless, changes in the composition of plant material ingested by the termites may affect what bacteria are deposited in the combs. If habitats offer different plant material as termite forage, this could also contribute to differences between years and across different sites, depending on whether such changes influence comb communities. Changes in the surrounding soil and mound microbiotas may also influence comb communities when combs come in contact with the soil, and these changes could be driven by environmental conditions, plant cover and composition, and macrofauna [37]. Differences in temperature, rainfall, or humidity may also affect soil microbiotas surrounding termite mounds. Data from the South African Weather Service (not shown) indicate that there were no discernible differences in maximum or minimum temperatures, daily rainfall, or humidity in the 12 months prior to the 2011 and 2013 collections. This suggests that climatic differences may not be the main reason for differences in comb community composition in our study; however, we cannot exclude that local conditions may affect the properties and growth conditions for bacteria communities within combs.

## Conclusions

Fungus comb microbiotas are generally very similar within termite genera and even species within years, but this pattern is disrupted over time by factors that our current knowledge of communities and environmental conditions cannot address. In contrast, and as expected, gut microbiotas were persistent over time and shaped by termite host species. Large proportions of the gut bacterial communities are shared with fungus combs, presumably transferred during fungus comb formation by termite deposits of primary faeces. Despite these gut community deposits to fungus comb communities, the latter harboured relatively small proportions of bacteria families present in termite guts, probably because fungus combs are more exposed and potentially receive a continuous influx of bacteria from the surroundings. The possible functions of fungus comb bacterial communities remain unknown, and future analyses of their metabolic activity will hopefully allow establishment of possible symbiotic roles.

## Electronic supplementary material

ESM 1 Figure S1. Phylogenetic analysis placing the 14 *Odontotermes* COII sequences in a phylogeny of available sequences from GenBank. Five of our samples grouped with *O.* cf. *badius*, while the remaining samples (*Odontotermes* sp.) group as a separate well-supported clade most closely related to *Odontotermes* sp. 1 and *Odontotermes latericeus*. Bootstrap support based on 500 pseudo-replicates under Neighbour-Joining conditions. Figure S2. Rarefaction curves of sequence depth generated with R. The curves represent the 33 termite comb samples and each curve shows the number of identified genus-level taxa as a function of the number of sequenced reads after filtering. The three samples where more sequences might have covered more bacterial genera are labelled. Figure S3. Phylum-level abundance distributions of reads across the 33 fungus comb samples. The heatmap is divided in three parts according to termite genera and year of collection is given at the top. A coloured logarithmic scale of phylum-level relative abundances is also provided. Figure S4. PCoA analyses visualising Bray-Curtis distances between samples originating from the three termite genera with sampling locations indicated next to the comb symbols (E = Experimental Farm, M = Mookgophong, R = RNC Farm, and L = Lajuma). A) 14 *Odontotermes* combs, solid circles are *O*. cf. *badius* and open circles are *Odontotermes* sp., B) 10 *M. natalensis* combs, and (C) 9 *Microtermes* sp. combs. The dashed lines indicate the splits between years of sampling. Figure S5. Relative abundances of the top 100 genus-level taxa that were on average most abundant across all fungus comb samples. On the horizontal axis are termite species and colony of origin, while the vertical axis presents bacterial taxa at the phylum, family and genus levels. The heatmap is divided in three parts according to the termite genera, where the year of sample collection is indicated at the top. The coloured logarithmic scale represents bacterial genus-level relative abundances within each microbiota. Figure S6. Rarefaction curves of sequence depth generated with R for the 25 gut samples. Each curve shows the number of identified OTUs as a function of the number of sequenced reads after filtering. Figure S7. Phylum-level abundance distributions of reads across the 25 gut samples. The heatmap is divided in three parts according to termite genera, and the bacterial families are listed from the most abundant at the top. A coloured logarithmic scale of phylum-level relative abundances is also provided. Figure S8. Relative abundances of the top 100 genus-level taxa that were on average most abundant across all gut samples. On the horizontal axis are termite species and colony of origin, while the vertical axis presents the bacterial genera. The heatmap is divided in three parts according to the termite genera. The coloured logarithmic scale represents bacterial genus-level relative abundances within each microbiota. (PDF 3.83 mb)

ESM 2 (XLSX 9.24 kb)

ESM 3 (XLSX 1.15 mb)

ESM 4 (XLSX 1.42 mb)

ESM 5 (XLSX 13.3 kb)
