# Supplementary figures and images for: Bacterial communities in termite fungus combs are comprised of consistent gut deposits and contributions from the environment
Source: Microb Ecol. 2015 Oct 30;71:207–20. doi: 10.1007/s00248-015-0692-6 (PMC4686563; doi:10.1007/s00248-015-0692-6)

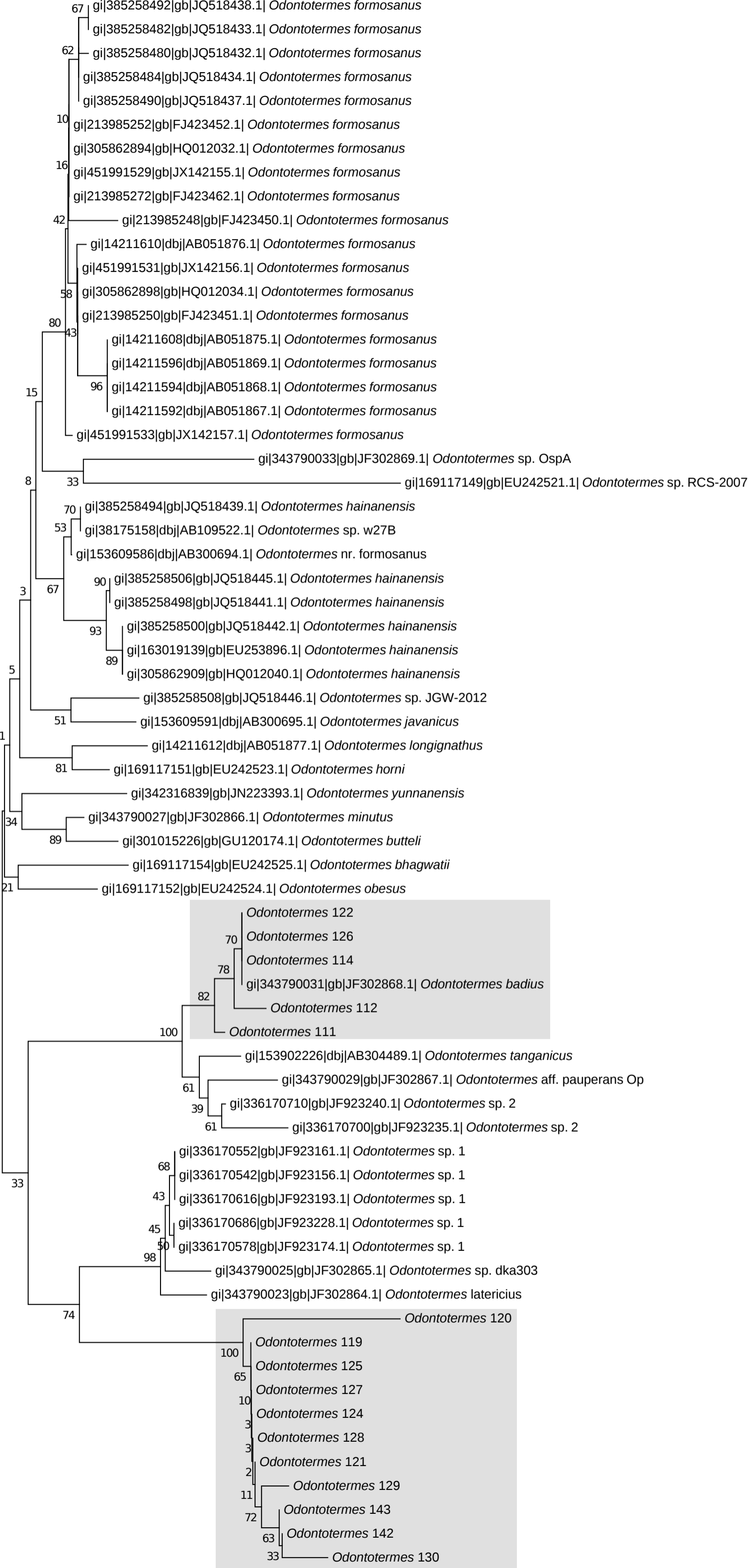

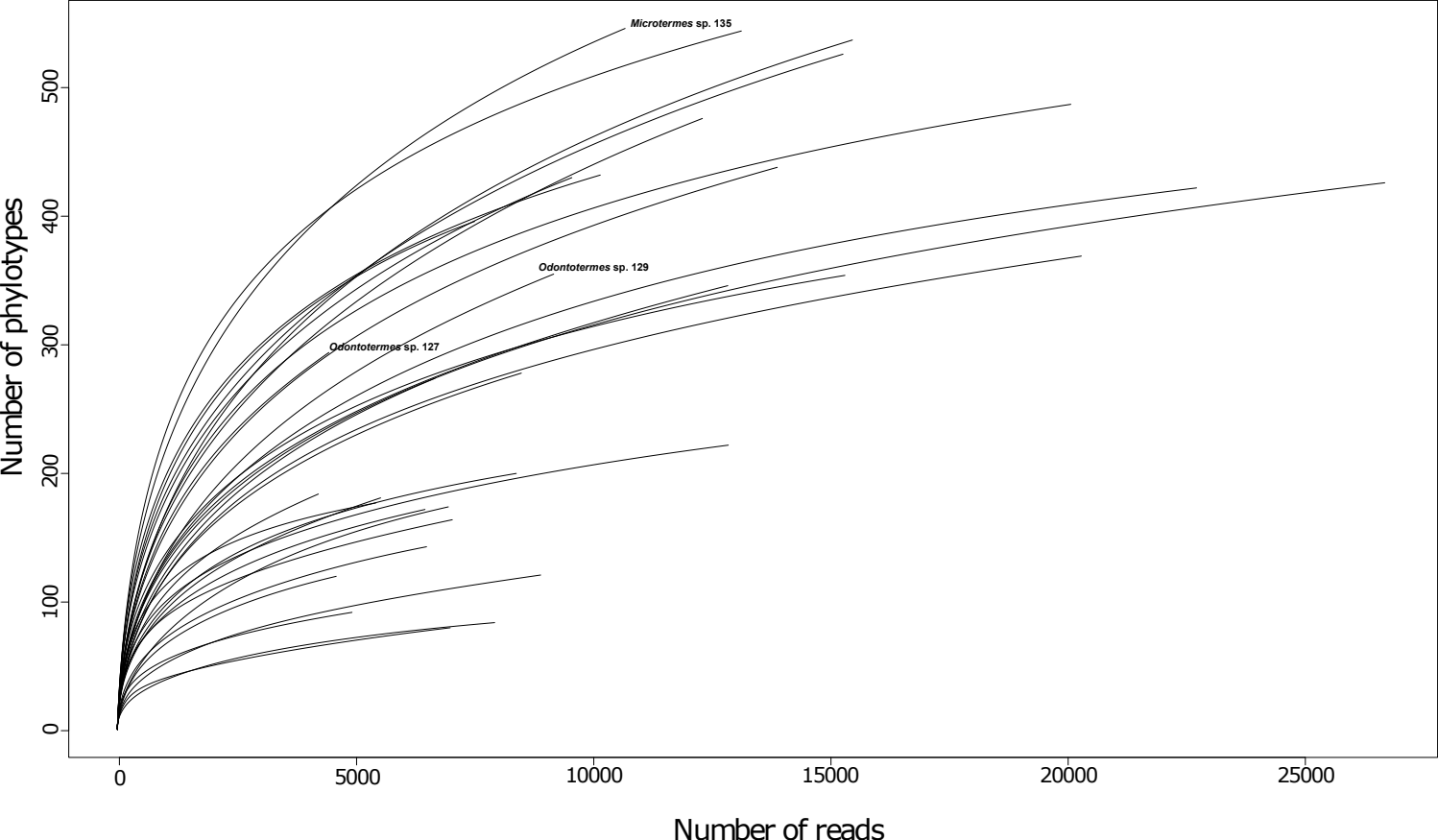

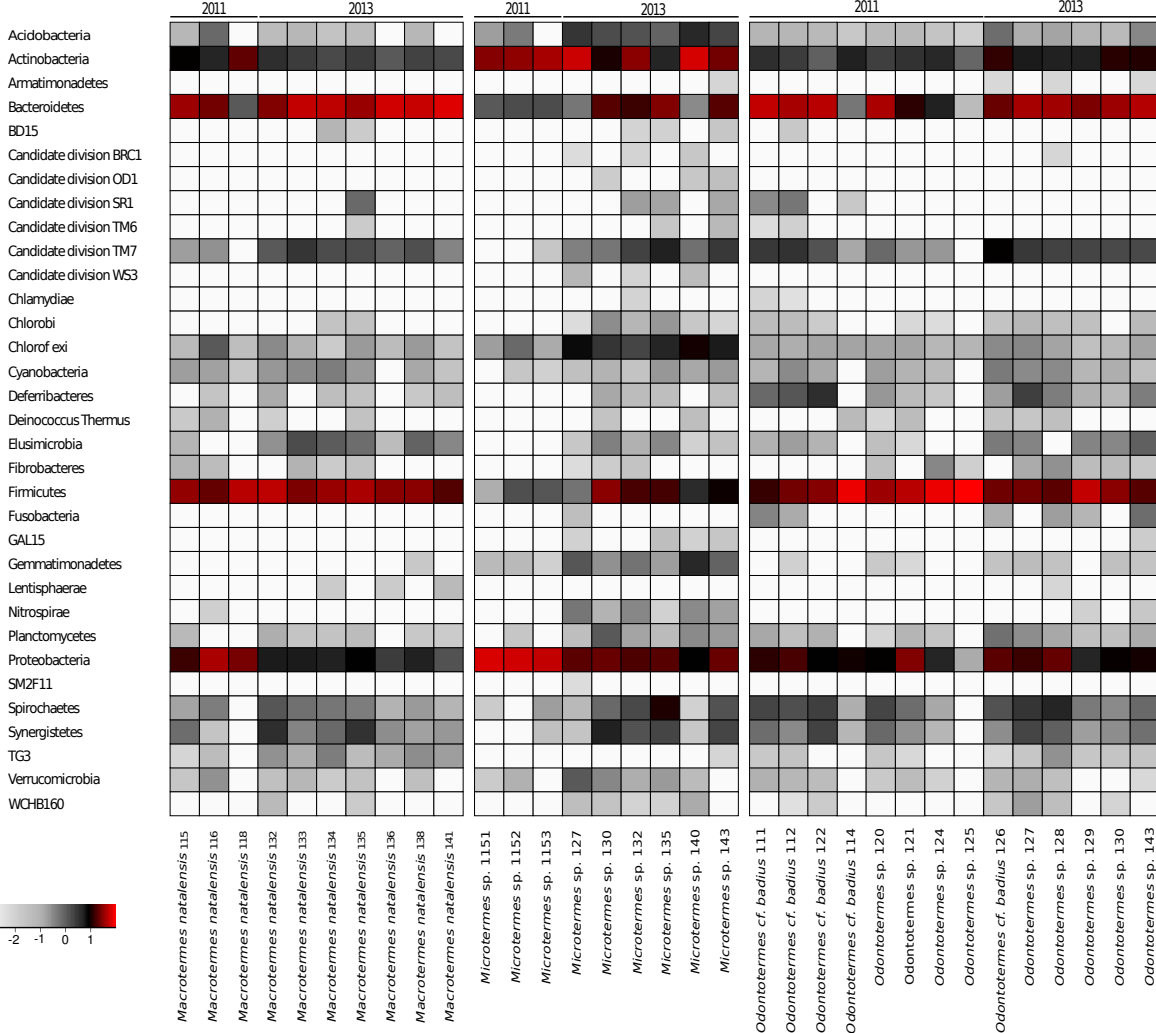

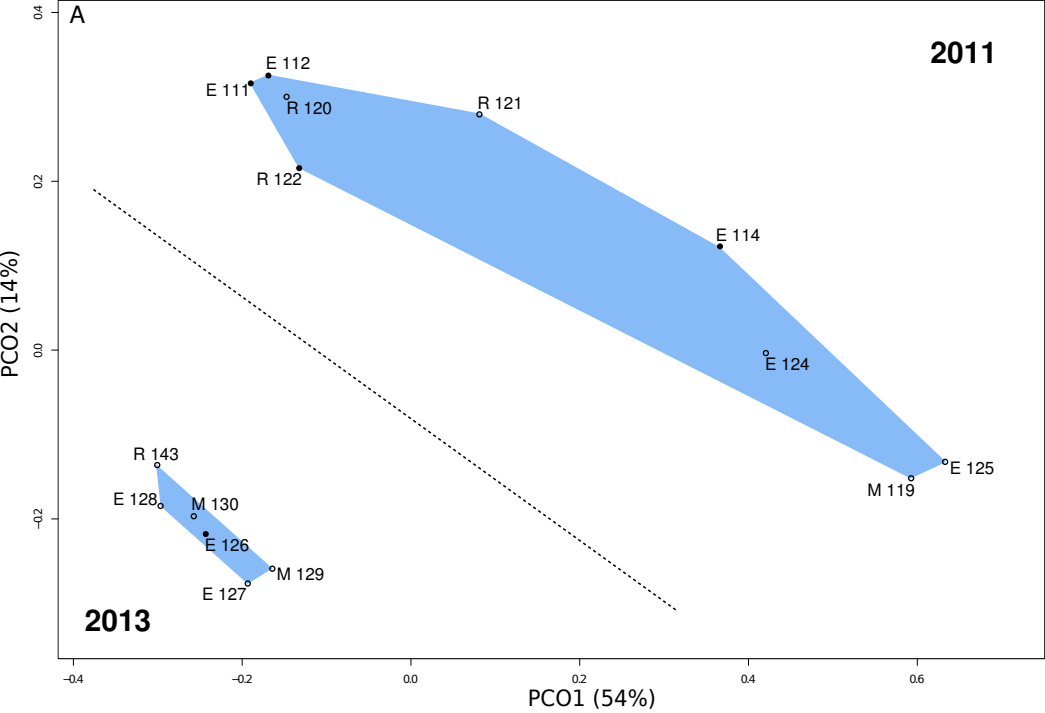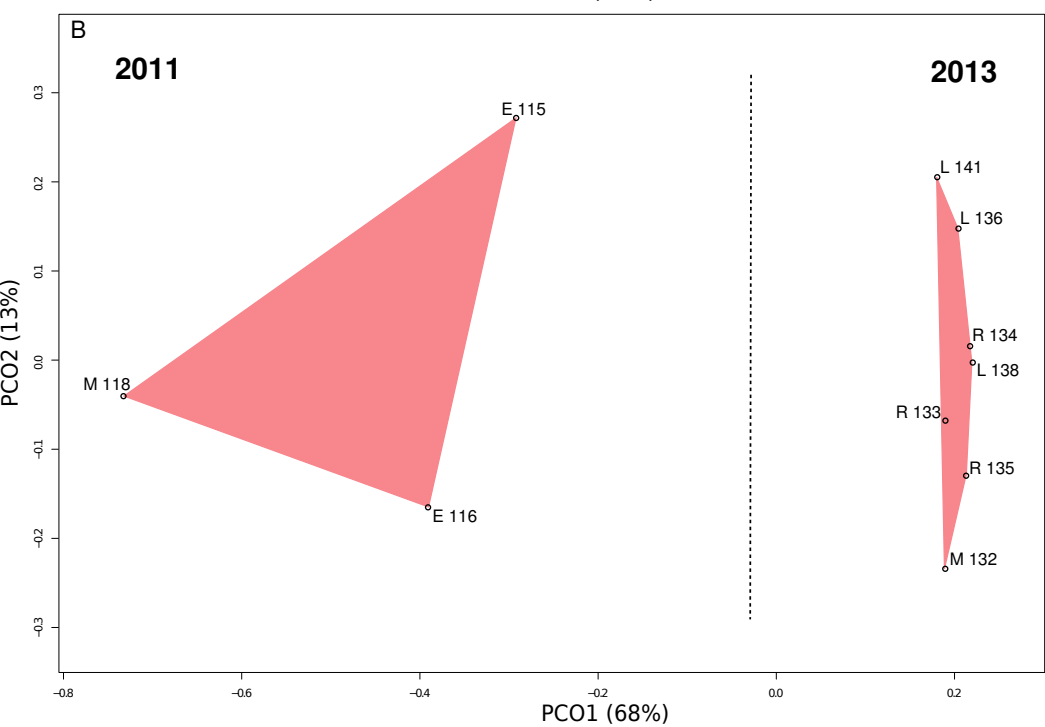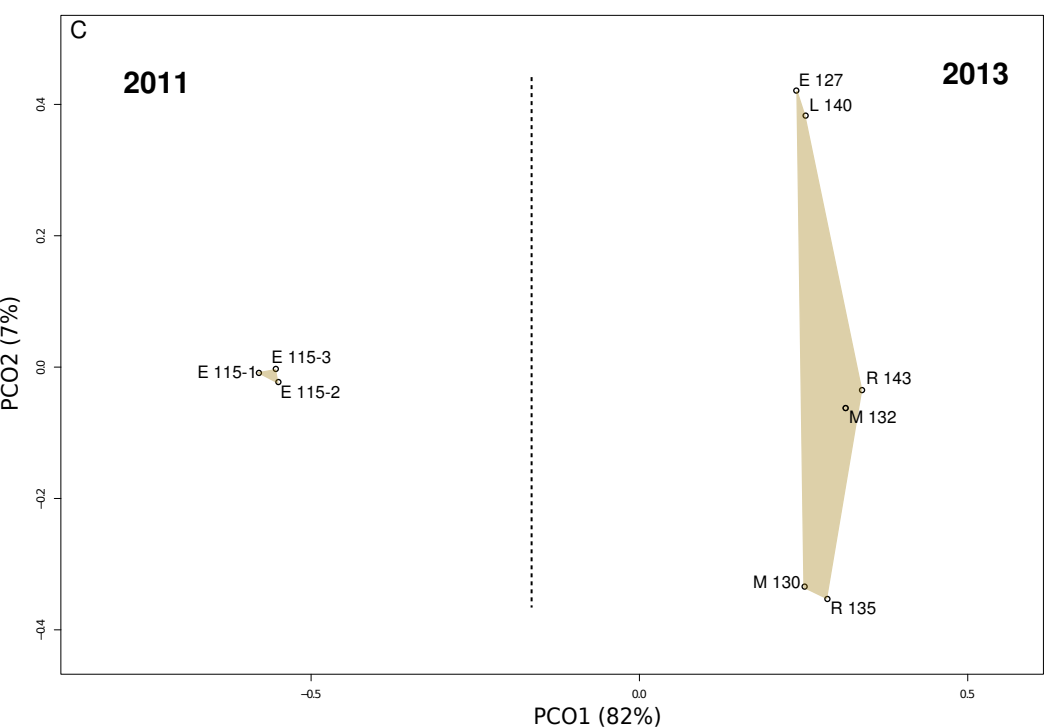



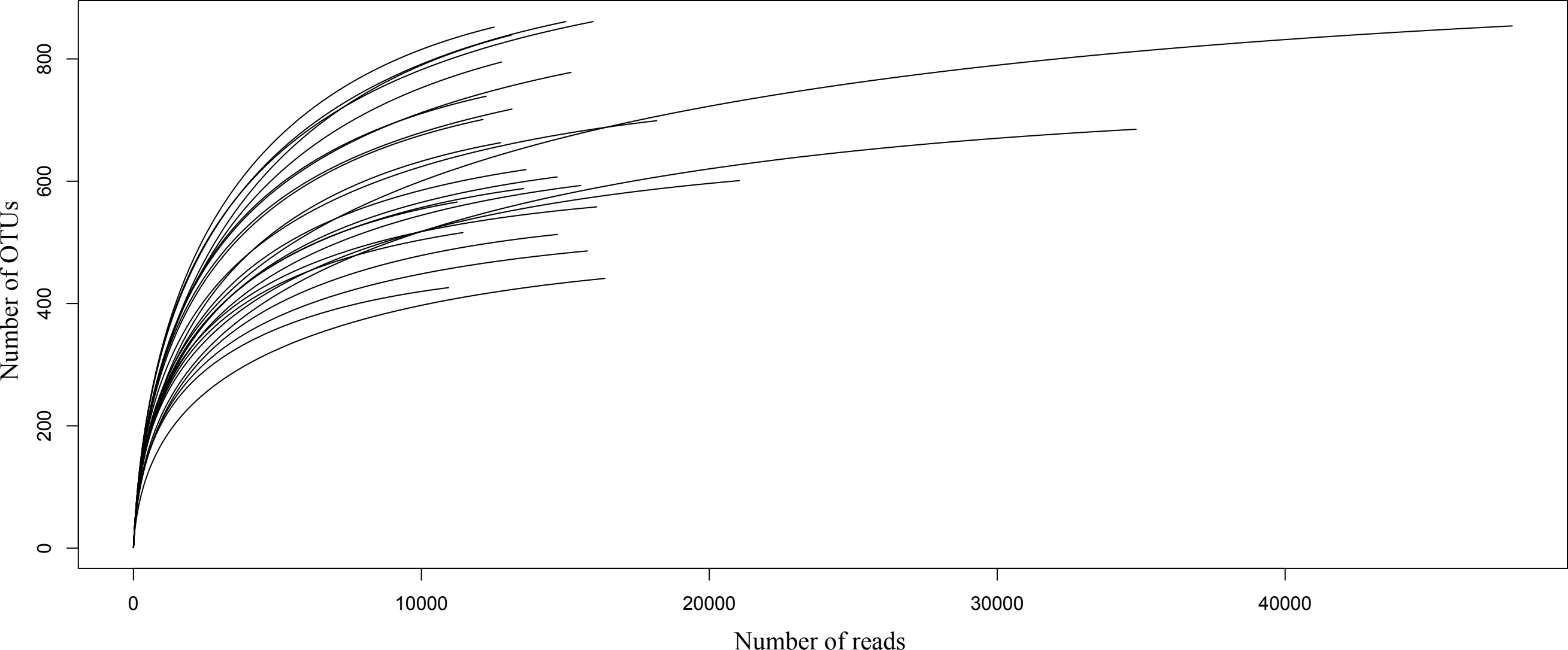

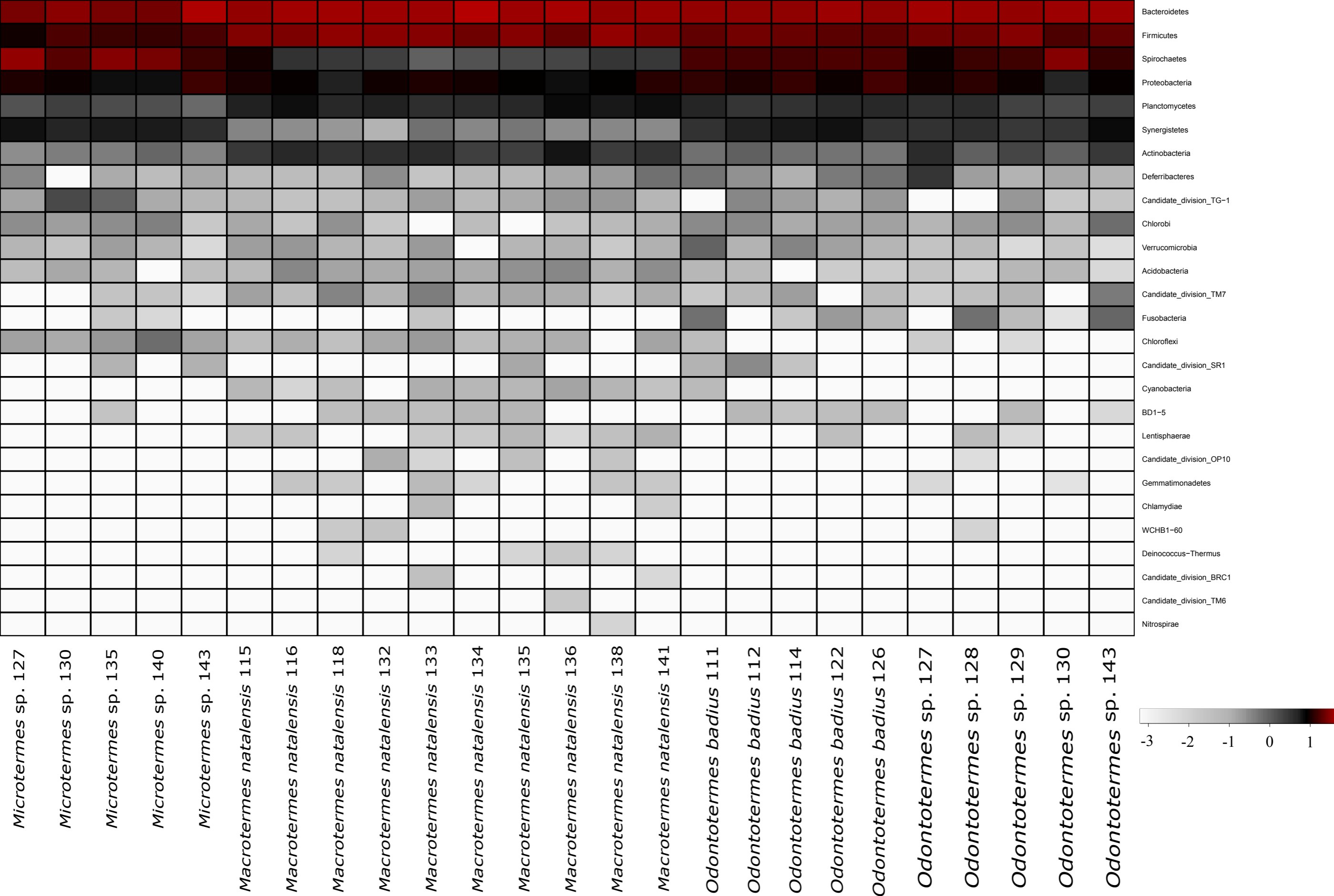

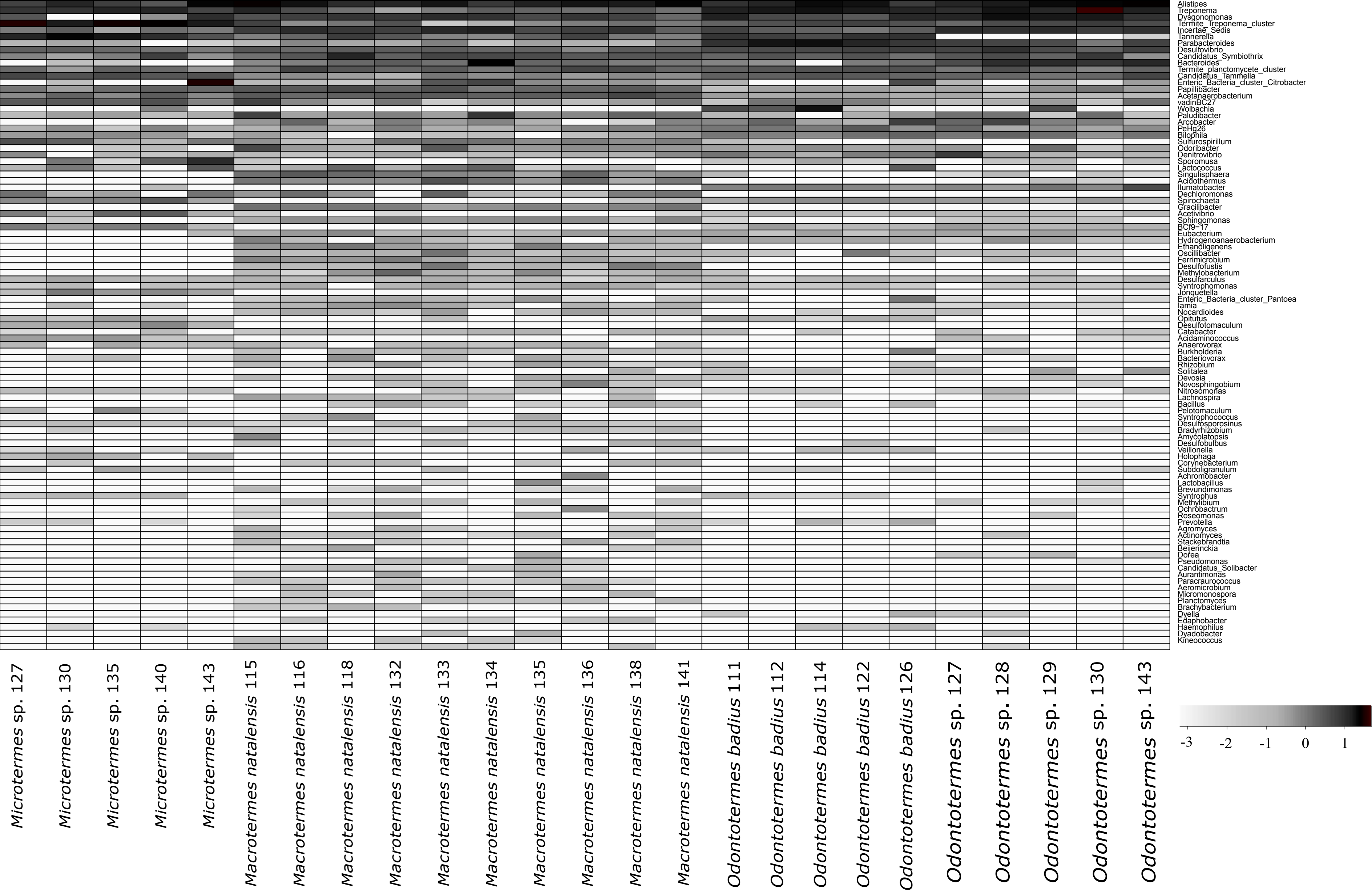

Supplement: Supplementary file 1 — Figure S1. Phylogenetic analysis placing the 14 Odontotermes COII sequences in a phylogeny of available sequences from GenBank. Five of our samples grouped with O. cf. badius, while the remaining samples (Odontotermes sp.) group as a separate well-supported clade most closely related to Odontotermes sp. 1 and Odontotermes latericeus. Bootstrap support based on 500 pseudo-replicates under Neighbour-Joining conditions. Figure S2. Rarefaction curves of sequence depth generated with R. The curves represent the 33 termite comb samples and each curve shows the number of identified genus-level taxa as a function of the number of sequenced reads after filtering. The three samples where more sequences might have covered more bacterial genera are labelled. Figure S3. Phylum-level abundance distributions of reads across the 33 fungus comb samples. The heatmap is divided in three parts according to termite genera and year of collection is given at the top. A coloured logarithmic scale of phylum-level relative abundances is also provided. Figure S4. PCoA analyses visualising Bray-Curtis distances between samples originating from the three termite genera with sampling locations indicated next to the comb symbols (E = Experimental Farm, M = Mookgophong, R = RNC Farm, and L = Lajuma). A) 14 Odontotermes combs, solid circles are O. cf. badius and open circles are Odontotermes sp., B) 10 M. natalensis combs, and (C) 9 Microtermes sp. combs. The dashed lines indicate the splits between years of sampling. Figure S5. Relative abundances of the top 100 genus-level taxa that were on average most abundant across all fungus comb samples. On the horizontal axis are termite species and colony of origin, while the vertical axis presents bacterial taxa at the phylum, family and genus levels. The heatmap is divided in three parts according to the termite genera, where the year of sample collection is indicated at the top. The coloured logarithmic scale represents bacterial genus-level relative a [file 248_2015_692_MOESM1_ESM.pdf]
